# Normative data for the EORTC QLQ-C30 from the Austrian general population

**DOI:** 10.1186/s12955-020-01524-8

**Published:** 2020-08-12

**Authors:** Jens Lehmann, Johannes M. Giesinger, Sandra Nolte, Monika Sztankay, Lisa M. Wintner, Gregor Liegl, Matthias Rose, Bernhard Holzner, on behalf of the EORTC Quality of Life Group

**Affiliations:** 1 University Hospital of Psychiatry II, Medical University of Innsbruck, Anichstrasse 35, 6020 Innsbruck, Austria; 2 Division of Psychosomatic Medicine, Medical Department, Charité - Universitätsmedizin Berlin, Corporate Member of Freie Universität Berlin, Humboldt-Universität uu Berlin, and Berlin Institute of Health, Berlin, Germany; 3 School of Health and Social Development, Population Health Strategic Research Centre, Deakin University, Burwood, VIC Australia

**Keywords:** Normative data, EORTC QLQ-C30, Quality of life, Oncology, Patient-reported outcome measures, Austria, General population

## Abstract

**Background:**

The European Organisation for Research and Treatment of Cancer (EORTC) QLQ-C30 is a widely used cancer-specific questionnaire assessing 15 domains of health-related quality of life (HRQoL). Our aim was to facilitate the interpretation of scores on this questionnaire by providing Austrian normative data based on a general population sample.

**Methods:**

The calculation of normative data was based on the EORTC QLQ-C30 data collected from an Austrian general population sample that was part of an international online panel study on the development of European normative data. Data reported herein were stratified and weighted by age and sex. Normative data were calculated for all 15 HRQoL domains of the EORTC QLQ-C30. For precise predictions of EORTC QLQ-C30 scores, a regression model based on sex, age and the presence of health conditions was built.

**Results:**

The Austrian sample comprised 1002 Austrian participants (50.1% female, 51.4% when weighted by age and sex based on United Nation statistics). The mean age was 53.7 years (weighted: 47.7 years) and 53.6% (weighted: 47.4%) reported at least one health condition. Men reported better physical (Cohen’s *d* = 0.17) and emotional (Cohen’s *d* = 0.17) functioning as well as less fatigue (Cohen’s *d* = 0.18) and insomnia (Cohen’s *d* = 0.25) compared with women. Younger individuals (< 40 years) reported less dyspnea (Cohen’s *d* = 0.61) and pain (Cohen’s *d* = 0.51), whereas older individuals (≥60 years) reported better emotional functioning (Cohen’s *d* = 0.55).

**Conclusions:**

We present Austrian normative data for the EORTC QLQ-C30. Differences by age and sex are mostly in line with the findings of other European normative studies. The Austrian population sample shows higher HRQoL and lower morbidity compared with other European countries. The normative data in this study will facilitate the interpretation of EORTC QLQ-C30 scores in oncological practice and research at a national and international level (including cross-cultural comparisons).

## Background

Patient-reported outcomes (PROs) are the gold standard of evaluating the impact of cancer and its treatment from the patient’s perspective. The importance of PROs is reflected in their widespread use as study endpoints in cancer clinical trials [1, 2] and in the steadily increasing integration of routine PRO assessments in daily clinical practice [3, 4]. Regulatory authorities such as the United States Food and Drug Administration [5] and the European Medicines Agency [6] have published guidance documents to foster the collection of high-quality PRO data in clinical trials. Guidelines from international associations such as the European Organisation for Research and Treatment of Cancer (EORTC) [7] and the International Society for Quality of Life Research [8] deal with the integration of PRO measures into clinical routine, and provide information on how to collect, process, and use PRO data. In the present study, we calculate reference values based on data assessed with EORTC QLQ-C30 [9], a questionnaire that is among the most widely used PRO measures in cancer clinical trials [10–13]. This questionnaire covers important functional health domains (e.g., physical and emotional functioning) and key cancer symptoms such as fatigue, pain, and nausea.

In the literature, different methods have been proposed to support the interpretation of results from PRO measures, analyzing not only individual patients and patient groups at a single time point, but also group differences and changes over time. One approach is to use minimally important differences to assess or compare changes in PRO results at the group or patient level [14]. To interpret scores obtained at a single time point, thresholds for clinical importance can be used [15]. Another important approach to interpret PRO data are normative data, if comparisons are to be made with, for example, a specific disease group or the general population on an individual- or group-level. When using normative data, it is important to note that these can vary considerably between countries [16–18]. Focusing on country-specific values allows for more precise interpretation, which becomes even more accurate when regression models that take age and sex distributions into account are used to generate PRO predictions.

The EORTC Quality of Life Group conducted a large-scale international panel study that collected normative data for the EORTC QLQ-C30 for 13 European countries, Canada, and the United States [19]. While the EORTC QLQ-C30 has widely been used in studies in Austria (e.g., [20–22]), no normative studies have so far been published. In the current study, we present age- and sex-specific normative data for the 15 health domains of the EORTC QLQ-C30 from the Austrian general population. In addition, we provide a regression model defined by age, sex and the presence of health conditions to calculate normative data for specific groups.

## Methods

### Sampling

As part of an international study conducted by Nolte et al. [19], data from the Austrian general population were collected by GfK SE (https://www.gfk.com/), a panel research company, which contacted panel members who had voluntarily registered and agreed to participate in panel-based studies. An equal number of participants was recruited for each sex and age group (male/female; 18–39, 40–49, 50–59, 60–69, ≥70 years). Once a quota was met for a specific group, the recruitment for this group was stopped. Response rates to panel studies conducted by GfK are between 75 and 90% as participants are registered voluntarily and usually participate when contacted.

### EORTC QLQ-C30

The EORTC QLQ-C30 consists of 30 items covering five functioning scales (physical, social, emotional, role, and cognitive), nine symptom scales (fatigue, nausea/vomiting, pain, dyspnea, sleep disturbances, appetite loss, constipation, diarrhea, and financial impact), and a global health status scale [9]. Referring to a recall period of one-week (except for physical function, which does not refer to a recall period at all), patients indicate their answers on a 4-point Likert scale. Linear converted scale scores range from 0 to 100. Higher scores on the functioning scales and on the global health status scale indicate better functioning, whereas higher scores on the symptom scales indicate greater symptom burden (for details on the scoring and scale structure see [23]). The EORTC QLQ-C30 has been validated in a large European samples, and has been widely used in German-speaking patients with cancer and the general population in Germany [18, 19, 24].

### Statistical analysis

Weights were established following Nolte et al. [19] and were based on official population distribution statistics published by the United Nations [25]. We weighted the responses to correct for over- or under-representation of sex- and age-stratified subgroups [26]. We report normative values as means and standard deviations for each subgroup. In addition, we established a regression model to predict EORTC QLQ-C30 scores with the following independent predictors: sex, age, age^2^, a two-way interaction age by sex, and the presence of health conditions (none or at least one health condition). We chose the predictors as they are linked to HRQoL and have been applied in previous studies [27–29]. The basic model can be expressed as follows: Intercept + Sex (male: 0; female: 1) + Age + Age^2^ + Interaction of Age and Sex (age * sex) + Presence of Health Conditions (none: 0; at least one: 1).

## Results

### Sample

In April and May 2017 we collected online survey data from 1002 individuals from the Austrian general population with an equal distribution of participants in the predefined age and sex groups. Participants were on average 53.7 years old (47.7 years when weighted) and 53.6% of participants (47.4% when weighted) reported at least one health condition. Some participants reported more than one health condition: 14.1% (11.9% when weighted) reported two, and 9.4% (7.6% when weighted) reported three or more health conditions. The most frequently reported health conditions were chronic pain 25.1% (21.6% when weighted) and arthritis 11.3% (9.1% when weighted). Regarding relationship status, the percentage of respondents in our sample who said that they were in a long-term relationship was 69.6 and 63.9% when weighted. The majority of respondents (87% or 87.8% when weighted) reported at least some post-compulsory education and 35% (or 36% when weighted) obtained a university degree (i.e., bachelors or higher). The full descriptive data are reported in Table 1.

**Table 1:** Sample characteristics (*N* = 1002)

		Unweighted data	Weighted data
Sex N (%)	Female	502 (50.1%)	515 (51.4%)
	Male	500 (49.9%)	487 (48.6%)
Age (years)	M (SD)	53.7 (15.3)	47.7 (17.3)
	Median (IQR)	54 (43–67)	48 (33–62)
Education^a^ N (%)	Less than compulsory (no or some primary education)	1 (0.1%)	3 (0.3%)
	Compulsory (about 9 years of schooling)	128 (12.9%)	118 (11.9%)
	Some post-compulsory (above 9 years of schooling without reaching university entrance certificate)	234 (23.6%)	221 (22.3%)
	Post-compulsory below university	281 (28.4%)	292 (29.5%)
	College (bachelor’s or equivalent level)	105 (10.6%)	120 (12.1%)
	University degree (Master, Doc, or equivalent)	242 (24.4%)	237 (23.9%)
	Prefer not to answer ^a^	11	11
Marital status^a^ N (%)	Single	159 (16.0%)	242 (24.4%)
	Married (or in a steady relationship)	692 (69.6%)	633 (63.9%)
	Separated / divorced / widowed	143 (14.4%)	116 (11.7%)
	Prefer not to answer ^a^	8	12
Employment status^a^ N (%)	Employed full time	357 (35.8%)	396 (39.8%)
	Employed part time	86 (8.6%)	95 (9.5%)
	Homemaker	17 (1.7%)	15 (1.5%)
	Student	30 (3%)	78 (7.8%)
	Unemployed	22 (2.2%)	28 (2.8%)
	Retired	361 (36.2%)	262 (26.3%)
	Self-employed	94 (9.4%)	91 (9.1%)
	Other	30 (3%)	31 (3.1%)
	Prefer not to answer ^a^	5	6
Health status^a,b^ N (%)	No health condition	444 (46.4%)	506 (52.6%)
	At least one health condition	513 (53.6%)	456 (47.4%)
	Chronic pain	240 (25.1%)	208 (21.6%)
	Heart disease	60 (6.3%)	45 (4.7%)
	Cancer	28 (2.9%)	23 (2.4%)
	Depression	61 (6.4%)	61 (6.3%)
	COPD	33 (3.4%)	26 (2.7%)
	Arthritis	108 (11.3%)	87 (9.1%)
	Diabetes	62 (6.5%)	47 (4.9%)
	Asthma	33 (3.5%)	34 (3.5%)
	Anxiety disorder	24 (2.5%)	24 (2.5%)
	Obesity	65 (6.8%)	56 (5.9%)
	Drug/alcohol use disorder	5 (0.5%)	5 (0.5%)
	Other	162 (16.9%)	143 (14.8%)
	Prefer not to answer	37	34
	Missing	8	6

*M* mean, *SD* standard deviation, *IQR* interquartile range,

^a^ For calculating percentages, the category “prefer not to answer” was treated as missing data,

^b^ The sum of all health conditions is larger than the sample size as respondents could choose multiple

### Normative data for the EORTC QLQ-C30

Table 2 shows a summary of mean scores and standard deviations for the EORTC QLQ-C30 across all analyzed sex/age groups. Men and women differed in several domains; the three largest differences were observed for the scales measuring insomnia (22.3 points for women vs. 16.5 points for men, Cohen’s *d* = 0.25), fatigue (26.1 points for women vs. 22.0 points for men, Cohen’s *d* = 0.18), and emotional functioning (76.3 points for women vs. 80.1 points for men, Cohen’s *d* = 0.17).

**Table 2:** EORTC QLQ-C30 normative data for the Austrian general population stratified by age and sex (*N =* 1002)

		Total	Females							Males						
			All	18–29	30–39	40–49	50–59	60–69	≥70	All	18–29	30–39	40–49	50–59	60–69	≥70
Physical functioning	M	**89.72**	88.59	90.85	93.33	88.98	90.47	86.93	81.27	90.92	93.96	89.86	92.19	91.13	92.27	84.00
	SD	**13.89**	14.43	9.69	10.81	14.26	13.81	16.02	17.98	13.22	6.56	12.70	12.57	14.48	12.17	18.76
Role functioning	M	**88.92**	87.71	91.09	92.98	86.63	87.67	86.63	81.50	90.19	94.79	93.24	89.39	89.00	87.83	83.83
	SD	**20.35**	21.90	18.89	16.01	25.19	19.64	23.28	25.40	18.50	10.62	15.20	20.69	20.97	20.15	21.96
Emotional functioning	M	**78.13**	76.31	74.03	71.78	71.12	77.50	80.94	83.08	80.06	78.65	75.60	78.11	78.67	84.58	88.00
	SD	**22.25**	22.85	26.18	24.73	23.69	20.55	18.51	19.08	21.46	24.85	21.91	23.81	21.58	14.62	12.92
Cognitive functioning	M	**89.06**	88.83	87.98	91.81	88.61	90.00	88.78	86.50	89.31	89.58	91.79	88.38	90.50	86.83	87.83
	SD	**17.76**	17.39	21.47	14.10	16.33	15.90	17.23	17.20	18.16	22.83	14.46	20.97	15.95	16.00	13.40
Social functioning	M	**92.23**	92.60	95.35	94.15	89.93	91.50	92.08	92.17	91.84	90.63	92.51	91.41	90.83	94.00	93.00
	SD	**17.14**	15.95	9.78	13.14	19.32	18.13	18.55	15.80	18.33	20.05	18.18	18.66	19.60	13.97	17.16
Global health/QoL	M	**75.65**	73.98	75.00	77.34	71.37	74.42	74.59	71.75	77.41	80.73	77.78	74.83	76.25	77.17	76.75
	SD	**20.02**	20.64	19.20	16.61	21.73	21.70	22.15	21.94	19.20	20.01	19.98	17.59	18.79	17.33	20.99
Fatigue	M	**24.12**	26.13	28.17	23.98	28.71	23.44	24.86	26.56	22.00	23.61	25.44	22.67	20.44	18.67	19.44
	SD	**22.72**	23.72	19.61	22.64	25.56	25.58	25.02	24.37	21.43	20.84	22.21	23.28	23.00	18.54	18.83
Nausea/vomiting	M	**1.99**	2.38	5.04	2.05	2.48	1.33	1.82	1.00	1.57	2.60	1.21	2.02	0.50	1.67	1.00
	SD	**8.29**	9.63	16.37	9.46	7.60	5.65	6.21	4.63	6.56	8.49	5.20	7.64	3.71	7.33	4.64
Pain	M	**19.96**	21.28	14.34	15.20	19.64	24.83	24.59	29.83	18.56	13.54	12.56	20.88	21.17	23.17	23.17
	SD	**24.33**	25.37	18.97	18.93	25.12	26.77	26.70	30.65	23.12	20.27	19.66	24.34	23.21	23.16	26.96
Dyspnea	M	**10.89**	12.15	3.88	7.60	15.51	10.67	16.83	20.00	9.56	5.21	8.70	8.08	11.67	11.33	15.33
	SD	**20.57**	20.89	10.74	16.65	21.91	20.05	24.85	25.08	20.16	14.76	17.75	19.70	22.92	20.84	24.87
Insomnia	M	**20.01**	23.33	19.38	14.04	25.41	28.00	31.02	23.67	16.49	9.37	11.11	21.89	21.67	17.33	19.67
	SD	**27.84**	30.27	27.25	23.47	29.89	33.77	33.51	31.18	24.55	19.15	21.12	27.02	26.98	23.52	26.91
Appetite loss	M	**4.38**	5.88	9.30	5.26	3.63	4.67	5.61	6.00	2.79	2.08	1.93	4.38	2.33	3.67	2.67
	SD	**16.09**	19.61	26.37	17.54	14.07	17.75	17.73	19.76	11.00	8.11	7.84	15.55	9.78	13.36	10.28
Constipation	M	**6.18**	7.76	5.43	9.36	9.57	4.33	6.60	11.33	4.50	2.08	4.83	3.70	5.00	4.33	8.67
	SD	**17.28**	19.95	19.02	23.31	20.74	13.95	17.70	22.84	13.72	8.11	14.31	12.51	15.27	12.27	19.37
Diarrhea	M	**7.54**	7.13	8.53	7.02	7.26	8.00	7.26	4.67	7.98	8.33	7.25	6.40	10.67	6.67	8.00
	SD	**18.66**	18.26	19.24	17.48	18.03	20.73	18.07	15.73	19.08	20.51	14.97	18.25	20.60	19.02	20.22
Financial difficulties	M	**5.05**	5.96	6.98	2.34	7.26	6.67	7.26	5.00	4.08	4.17	5.80	3.70	4.33	2.33	3.67
	SD	**17.56**	18.77	21.13	10.63	21.93	20.12	20.39	15.27	16.13	18.25	20.56	13.38	15.48	9.81	15.68

*M* Mean scores, *SD* standard deviation, *QoL* quality of life

The three largest differences between age groups were observed for the scales measuring dyspnea (4.6 points for those aged 18–29 years vs. 18.1 points for those aged ≥70 years, Cohen’s *d* = 0.71), pain (13.9 points for those aged 18–29 years vs. 27.1 points for those aged ≥70 years, Cohen’s *d* = 0.54), and insomnia (12.6 points for those aged 30–39 vs. 24.8 points for those aged 50–59 years, Cohen’s *d* = 0.46). See Table 3 for the normative data stratified by age group.

**Table 3:** EORTC QLQ-C30 normative data for the Austrian general population stratified by age (*N =* 1002)

		Total	18–29	30–39	40–49	50–59	60–69	≥70
Physical functioning	M	**89.72**	92.44	91.59	90.58	90.80	89.48	82.39
	SD	**13.89**	8.37	11.89	13.50	14.11	14.50	18.30
Role functioning	M	**88.92**	92.98	93.11	88.02	88.33	87.21	82.46
	SD	**20.35**	15.32	15.56	23.02	20.26	21.76	24.00
Emotional functioning	M	**78.13**	76.38	73.70	74.62	78.08	82.68	85.11
	SD	**22.25**	25.56	23.36	23.94	21.01	16.80	16.95
Cognitive functioning	M	**89.06**	88.80	91.80	88.50	90.25	87.85	87.05
	SD	**17.76**	22.14	14.23	18.74	15.88	16.61	15.72
Social functioning	M	**92.23**	92.94	93.33	90.68	91.17	93.00	92.51
	SD	**17.14**	16.01	15.85	18.95	18.83	16.48	16.32
Global health/QoL	M	**75.65**	77.92	77.56	73.10	75.33	75.82	73.81
	SD	**20.02**	19.78	18.32	19.78	20.26	19.96	21.63
Fatigue	M	**24.12**	25.84	24.71	25.69	21.95	21.90	23.63
	SD	**22.72**	20.33	22.37	24.57	24.30	22.29	22.47
Nausea/vomiting	M	**1.99**	3.80	1.63	2.25	.92	1.74	1.00
	SD	**8.29**	12.99	7.61	7.60	4.79	6.74	4.62
Pain	M	**19.96**	13.93	13.88	20.26	23.00	23.91	27.09
	SD	**24.33**	19.60	19.28	24.67	25.05	24.98	29.29
Dyspnea	M	**10.89**	4.56	8.15	11.79	11.17	14.20	18.08
	SD	**20.57**	12.93	17.16	21.10	21.48	23.09	25.02
Insomnia	M	**20.01**	14.28	12.57	23.65	24.84	24.48	22.02
	SD	**27.84**	23.95	22.30	28.46	30.64	29.85	29.48
Appetite loss	M	**4.38**	5.62	3.59	4.00	3.50	4.68	4.63
	SD	**16.09**	19.64	13.62	14.79	14.34	15.76	16.57
Constipation	M	**6.18**	3.72	7.08	6.63	4.67	5.52	10.24
	SD	**17.28**	14.58	19.39	17.33	14.59	15.33	21.45
Diarrhea	M	**7.54**	8.43	7.13	6.83	9.33	6.98	6.04
	SD	**18.66**	19.85	16.22	18.09	20.65	18.45	17.73
Financial difficulties	M	**5.05**	5.54	4.08	5.48	5.50	4.90	4.45
	SD	**17.56**	19.72	16.43	18.19	17.94	16.34	15.40

*M* Mean scores, *SD* standard deviation, *QoL* quality of life

For functioning scales, ceiling effects (i.e. achieving the maximum score) were lowest for emotional functioning (25.1%, *n* = 252) and most prevalent for social functioning (76.3%, *n* = 765). Floor effects (i.e. achieving the minimum score) were observed most frequently in nausea/vomiting (92.1%, *n* = 923) and least common for fatigue (24.6%, *n* = 247).

### Regression model predicting EORTC QLQ-C30 scores

Table 4 shows the regression model predicting individual EORTC QLQ-C30 scores using the weighted normative data. This model predicts EORTC QLQ-C30 scores using the individual’s sex, age, and presence of health conditions. An easy to use spreadsheet for the calculation of predicted values for individuals and groups is available online (see Supplementary 1). For example, for a woman aged 59 years with at least one health condition, the predicted physical functioning score is calculated as follows: Intercept (84.533) + Sex (1 * 0.463) + Age (59 * 0.564) + Age^2^ (59 * 59 * − 0.007) + Interaction of Age and Sex (1 * 59 * − 0.053) + Presence of Health Conditions (1 * − 7.443) = 83.34.

**Table 4:** Regression model for predicting EORTC QLQ-C30 scores using age, sex, and the presence of health conditions

	Intercept	Sex	Age in years	Age in years (squared)	Age-by-sex	Health conditions (yes/no)
Physical functioning	84.533	0.463	0.564	-0.007	-0.053	-7.443
Role functioning	90.685	−3.946	0.378	−0.005	0.030	−11.821
Emotional functioning	83.923	−6.531	−0.272	0.006	0.062	−14.523
Cognitive functioning	81.214	−2.626	0.567	−0.005	0.053	−10.696
Social functioning	92.991	4.809	0.026	0.001	−0.084	−11.907
Global health/QoL	79.543	−5.945	0.156	− 0.001	0.064	−16.450
Fatigue	34.835	0.769	−0.631	0.004	0.054	15.793
Nausea/vomiting	7.393	3.556	−0.283	0.002	−0.058	3.455
Pain	8.274	−0.048	−0.020	0.001	0.040	20.817
Dyspnea	2.883	−4.231	−0.010	0.001	0.128	9.932
Insomnia	−4.711	8.358	0.588	−0.005	− 0.043	15.569
Appetite loss	15.318	9.242	−0.708	0.007	−0.128	7.158
Constipation	2.587	5.172	−0.064	0.001	−0.044	3.769
Diarrhea	6.984	3.574	−0.001	0.000	−0.095	4.532
Financial difficulties	6.042	0.449	−0.145	0.000	0.029	8.990

Coding is: sex (male = 0, female = 1), age in years (above 18), age by sex (age in years*sex); health conditions (no health conditions = 0, at least one health condition = 1); *QoL* quality of life

## Discussion

In this study, we present age- and sex-specific normative data for the EORTC QLQ-C30 from a sample of the Austrian general population. Men and women differed in several domains, most notably insomnia, fatigue, and emotional functioning. In general, men reported a higher functioning (except for social functioning) and less symptom burden (except for diarrhea) than women did. Older participants (≥70 years) reported higher symptom burden (e.g., pain and dyspnea) and lower physical and role functioning compared to younger participants. We observed both ceiling effects (for functioning scales) and floor effects (for symptom scales) for some scales of the EORTC QLQ-C30. However, floor and ceiling effects were not unexpected, considering that we administered a cancer-specific questionnaire to a general population sample.

A potential limitation of panel studies is that this method of data collection may be prone to under-representing specific groups of individuals (e.g., those who are older or less educated). Therefore, it should be considered whether the assessment of general population data collected via online surveys are truly representative. A comparison of our data with Statistics Austria’s 2017 report suggests that the data obtained from the online survey are representative in terms of most basic individual characteristics (age, sex, and marital status) [30]. For example, regarding the relationship status in our sample, 63.9% of participants stated to be in a long-term relationship. Statistic Austria reported a similar rate with 67.1% of Austrian adults being in a long-term relationship. The unemployment rate of the Austrian sample was 2.8% while the unemployment rate in the 2017 report of the Statistic Austria institute was 5.3% for individuals older than 20 years. Furthermore, the prevalence rates for common health conditions found in our data match other data on the Austrian population: The prevalence of self-reported chronic pain in our sample (21.8%) is close to the prevalence of chronic pain in Austria [30] as diagnosed by a doctor, which is 24.4% for chronic back pain and 18.5% for chronic neck pain. The percentage of participants with diabetes in our sample (6.3%) is in line with the 5–7% prevalence rate of diagnosed diabetes among adults estimated in the latest Austrian diabetes report published by the Austrian Health Ministry [31]. For cancer, the prevalence rate was 2.8%, which is only slightly lower than the 4.1% prevalence rate found in the latest Statistics Austria cancer report [32]. Notable differences of our data to the Statistics Austria data [30] were observed only regarding the level of education. More than one-third of individuals taking part in the online survey (35%) reported at least a university-level education while in the Statistic Austria data only 15% of the sample report a university or comparable degree. In our publication of international normative data for the EORTC CAT CORE (based on the international dataset), the relationship between education and HRQoL scales was investigated in depth [26]. Higher education (some post-compulsory vs. less than post-compulsory education) was linked to more favorable HRQoL scores. However, differences were of low practical relevance as indicated by the small effect sizes (all eta^2^ ≤ .015).

A strength of our study is the consistent data collection mode used by Nolte et al. [19], which allows comparison with other European normative data. Comparisons of HRQoL ratings across country borders can be made, providing insight into international differences [28, 33]. At the national level, our study offers two different approaches for interpreting EORTC QLQ-C30 data. First, the normative data allow interpretation and comparison for different age and sex groups. Second, the regression model permits the calculation of expected EORTC QLQ-C30 normative scores using sex, age, and the presence of health conditions. This regression model can be used to generate more precise predictions than those made through comparing different age and sex groups using normative data.

Compared with other score interpretation approaches, normative data, in particular when relying on regression models, offer the advantage of not reducing the amount of information. For example, in an alternative approach, thresholds for clinical importance are used to condense the information into severity categories (e.g., clinically important vs. not clinically important), which can ease interpretation but also decreases the amount of information conveyed. The data we present in this study can be used to compare HRQoL among patients with cancer and HRQoL in the general population, matched by sex and age group. Such comparisons can be useful at any stage of the cancer journey, as patients’ HRQoL is likely to be compromised at the time of diagnosis [34], and they can be used to determine whether cancer survivors return to population levels or whether problems and impairments persist [35, 36]. Ideally, such comparisons use country-specific normative data, which most accurately reflect the average level of HRQoL within a particular national context. Of course, normative data need a certain amount of currentness to be relevant. For the years to come, this publication can serve as a reference, but data should be updated in due time.

Several observed results merit in depth discussion. The sex differences observed across various domains (especially physical functioning) are in line with other normative studies on the EORTC QLQ-C30 that have reported differences by sex. In studies in Germany [18, 24], Slovenia [33], Denmark [34], Sweden [37], and the Netherlands [38], men tended to report higher functioning and lower symptom burden on most scales than women did. A similar pattern can be observed in the presented Austrian normative data, though both point differences and effect sizes were rather small. Our data allow for better interpretation of these differences among patients with cancer through the comparison of the magnitude of impairment in both groups of individuals (patients with cancer and members of the Austrian general population). As has been discussed previously [39], sex differences among patients with cancer do not necessarily reflect a sex-specific impact of disease or treatment; rather, these differences may reflect more general factors including sex-specific response styles that also affect the general population, as we observed in the present sample.

Regarding age, a European sample [19], as well as single-country studies in Denmark, Sweden, and Slovenia [33, 34, 37] report similar deterioration with lower physical and role functioning in older people (≥60 or ≥ 70 years) compared to younger people (< 60 or < 70 years) as found in our sample. Those studies also found emotional functioning to be independent of age [33, 34] or to even increase with age (≥60 years) [19, 37], showing a similar pattern of emotional functioning and age as observed in our sample. However, our sample differed from two study samples in Germany, which did not show an increase in emotional functioning with age and reported much higher disparity between age groups on the global health status scale, with a difference of up to 23 points (oldest vs. youngest group) in favor of younger people [18, 24].

Compared with the European sample reported by Nolte et al. [19], fewer respondents in our sample reported health conditions or diseases (36.6% in the European sample vs. 52.6% in our sample reported having no health conditions), and our sample showed higher functioning and lower symptom burden on most scales. According to the 2018 *EU Statistics on Income and Living Conditions*, the Austrian life expectancy is slightly above the EU average (81.3 years vs 80.6 years) and 71.7% of the Austrian adult population report a good or very good perceived health which ranks higher than most other European countries measured [40]. Both of these findings support our result of generally high HRQoL in terms of fewer health conditions and high functioning among the Austrian general population.

## Conclusions

The normative data for the EORTC QLQ-C30 generated in this study will ease and foster a more meaningful interpretation of scores obtained from patients with cancer and cancer survivors, allowing comparisons of patient-level and group-level data with the sex- and age-matched general population sample from Austria. Using our regression model, precise predictions for individuals based on sex, age, and presence of health conditions can be generated.

## Supplementary information

**Additional file 1.**

## Data Availability

The datasets analyzed in the study at hand are available upon reasonable request from the EORTC. Please use the Data Sharing form available through the EORTC website (https://www.eortc.org/data-sharing/).

## Abbreviations

EORTC: European Organisation for Research and Treatment of Cancer
GfK SE: Gesellschaft für Konsumforschung Societas Europaea
HRQoL: Health-Related Quality of Life
PRO: Patient-Reported Outcomes
